# Evaluating the attenuation of naphthenic acids in constructed wetland mesocosms planted with *Carex aquatilis*

**DOI:** 10.1007/s10661-023-11776-8

**Published:** 2023-09-19

**Authors:** Kaitlyn E. Trepanier, Ian J. Vander Meulen, Jason M. E. Ahad, John V. Headley, Dani Degenhardt

**Affiliations:** 1 Canadian Forest Service, Northern Forestry Centre, Natural Resources Canada, Edmonton, AB Canada; 2 Environment and Climate Change Canada, National Hydrology Research Centre, Saskatoon, SK Canada; 3 Department of Civil, Geological and Environmental Engineering, College of Engineering, University of Saskatchewan, Saskatoon, SK Canada; 4 Geological Survey of Canada, Natural Resources Canada, Québec, QC Canada

**Keywords:** oil sands process–affected water, Mesocosms, Naphthenic acid fraction compounds, Greenhouse

## Abstract

Surface oil sands mining and extraction in northern Alberta’s Athabasca oil sands region produce large volumes of oil sands process–affected water (OSPW). OSPW is a complex mixture containing major contaminant classes including trace metals, polycyclic aromatic hydrocarbons, and naphthenic acid fraction compounds (NAFCs). Naphthenic acids (NAs) are the primary organic toxicants in OSPW, and reducing their concentrations is a priority for oil sands companies. Previous evidence has shown that constructed wetland treatment systems (CWTSs) are capable of reducing the concentration of NAs and the toxicity of OSPW through bioremediation. In this study, we constructed greenhouse mesocosms with OSPW or lab process water (LPW) (i.e., water designed to mimic OSPW minus the NAFC content) with three treatments: (1) OSPW planted with *Carex aquatilis*; (2) OSPW, no plants; and (3) LPW, no plants. The OSPW–*C. aquatilis* treatment saw a significant reduction in NAFC concentrations in comparison to OSPW, no plant treatments, but both changed the distribution of the NAFCs in similar ways. Upon completion of the study, treatments with OSPW saw fewer high-molecular-weight NAs and an increase in the abundance of O_3_- and O_4_-containing formulae*.* Results from this study provide invaluable information on how constructed wetlands can be used in future remediation of OSPW in a way that previous studies were unable to achieve due to uncontrollable environmental factors in field experiments and the active, high-energy processes used in CWTSs pilot studies.

## Introduction 

The Athabasca oil sands region in northern Alberta contains the third largest oil reserve in the world, accounting for an estimated 165 billion barrels of extractable oil reserves with approximately 2.9 million barrels produced daily (Canadian Association of Petroleum Producers, 2022; Government of Canada, n.d.). Surface mining of bituminous sand produces large volumes of oil sands process–affected water (OSPW) and tailings as a by-product, wherein the OSPW in tailings ponds is recycled throughout the extraction process (AER (Alberta Energy Regulator), 2017). A “zero discharge” policy has been in place since 1993, resulting in the accumulation of over 1.3 billion m^3^ of tailings being stored in tailings ponds across the mineable oil sands region (AER, 2017; Canadian Association of Petroleum Producers, 2022; Government of Canada, n.d.). The free water in tailings ponds, or OSPW, are comprised of < 5% solids and contain varying amounts of salt, organic compounds, and trace metals (AER, 2017; Canadian Association of Petroleum Producers, 2022; Government of Canada, n.d.). OSPW presents considerable environmental and economic challenges due to the volume, complexity, and toxicity of the mixture (Allen, 2008). Within OSPW, there are several major classes of contaminants including naphthenic acids (NAs), polycyclic aromatic hydrocarbons (PAHs), BTEX (benzene, toluene, ethylbenzene, and xylenes), phenols, heavy metals, and ions (Alharbi et al., 2021; Allen, 2008; Cancelli & Gobas, 2020; McQueen et al., 2017a, b; Puttaswamy & Liber, 2012).

Of the many components in OSPW, naphthenic acid fractional compounds (NAFCs) are some of the primary organic toxicants of concern. This complex class of organic acids includes naphthenic acids (NAs), which have been consistently implicated as being particularly toxic (Hughes et al., 2017; Morandi et al., 2015). The general formula for NAs follows the format C_n_H_2n+Z_O_2_, where *n* is the number of carbon atoms and *z* is a negative even integer that indicates hydrogen deficiencies caused by the presence of a ring structure (Brown & Ulrich, 2015; Grewer et al., 2010; Headley et al., 2013; Wang et al., 2016; Xue et al., 2018). The broader compound class encompassed by NAFCs includes diverse organic acids that can be aromatic, contain sulfur or nitrogen heteroatoms, and may be polyoxygenated (e.g., *x* ≥ 2) (Brown & Ulrich, 2015; Grewer et al., 2010; Headley et al., 2013; Wang et al., 2016; Xue et al., 2018). NAs can be particularly resistant to biodegradation when functionalized with an ante-iso-(β)-alkyl substituted moiety, a ternary substitution other than β to the carboxylic group, or a methyl substitution on a ring structure is present (Quagraine et al., 2007). Additionally, NAFCs with more rings, higher molecular weights, and increased branching tend to be more resistant to biodegradation (Biryukova et al., 2007; Del Rio et al., 2006; Han et al., 2008). Molecular features and level of unsaturation can therefore be important determinants of environmental persistence of NAFCs.

As the most toxic component of NAFCs, NAs pose considerable toxicological risks for aquatic and terrestrial biota with acute and chronic adverse responses (Anderson et al., 2012; Bartlett et al., 2017; Hagen et al., 2014; Kavanagh et al., 2013). As an example, results of studies 24 and 25 showed that the lethal concentration that causes 50% mortality (LC_50_) estimated for wood frog (*Xenopus tropicalis*) tadpoles was 4.76 mg/L of technical grade NAs (C_10_H_18_O_2_) over a 24-hexposure period (Melvin & Trudeau, 2012), while fathead minnows (*Pimephales promelas*) exposed to 10 mg/L of NAs for 21 days spawned fewer eggs with an LC_50_ of 32.8 mg/L of NAs for minnow embryos (Kavanagh et al., 2012). Cattails (*Typha latifolia*) were also susceptible to acute toxic effects when exposed to OSPW-derived NAs at 60 mg/L (Armstrong et al., 2009). In OSPW, NA concentrations and compositions can vary depending on the extraction processes, type of ore, and age of OSPW (Allen, 2008; Holowenko et al., 2002), as NAs have an estimated half-life of approximately 13 years within tailings ponds (Han et al., 2009). Quantitation across sources and methods must be done sparingly; although NAs in OSPW can range between 20 and 120 mg/L, this depends on the source of OSPW (Ajaero et al., 2018; Cancelli & Gobas, 2022; Vander Meulen et al., 2021). Measurement methods must be kept consistent within study designs, as amounts of NAs and NAFCs will also vary between different quantification methods, whether using FTIR (Grewer et al., 2010; Ripmeester & Duford, 2019; Rogers et al., 2002), gas chromatography–mass spectrometry (Grewer et al., 2010), or various forms of liquid chromatography–mass spectrometry (Ajaero et al., 2018; Duncan et al., 2016; Headley et al., 2011a, b; Janfada et al., 2006).

Due to the persistence of NAFCs and its toxicity in OSPW, NAFCs and OSPW must be adequately degraded and toxicity reduced prior to the potential discharge back into the environment. The development of effective and efficient approaches for treating OSPW is required. Many active treatment techniques are being investigated (e.g., electrical or chemical oxidation, sequestration, and/or filtration techniques) for treating NAFCs in OSPW (Abdalrhman & El-din, 2020; Allen, 2008; Alpatova et al., 2014; Kim et al., 2012; Wang et al., 2016), but there have been few treatment strategies that have shown promise for large-scale and cost-effective implementation (Quinlan & Tam, 2015; Wang et al., 2016).

Constructed wetland treatment systems (CWTS) can facilitate the remediation of OSPW by degrading NAFCs through the microbial communities and wetland plants (Cancelli & Gobas, 2020; Hendrikse et al., 2018; McQueen et al., 2017a, b). Plants are incorporated into CWTS to directly or indirectly promote microbial activity by increasing aeration and accumulating organic matter (Allen, 2008; Phillips et al., 2010; Truu et al., 2015). Biodegradation of NAFCs by leveraging natural processes could be an effective method to reduce NAFC concentrations and related toxicity compared to resource- and energy-intensive chemical and physical approaches. It is imperative to understand the various parameters that affect the success of CWTS in reducing OSPW toxicity, particularly in northern areas like the Athabasca oil sands region with short growing seasons and cold winter temperatures (Allen, 2008; Truu et al., 2009).

Previous successful petroleum bioremediation projects have focused on the potential of wetland graminoids to degrade OSPW in a CWTS (Hendrikse et al., 2018; McQueen et al., 2017a, b; Simair et al., 2021). A variety of wetland graminoids (*Typha latifolia*, *Phragmites australis*, and *Scirpus acutus*) were able to selectively enhance the dissipation of nonionized NA compounds, effectively reducing the toxicity of NAs in the system over 30 days (Armstrong et al., 2009). Slender wheatgrass (*Elymus trachycaulus*) seedlings grown in substrate saturated with OSPW were found to directly uptake five types of NAs that represent aliphatic, single-ring, and three-ring diamondoid NA classes (Alberts et al., 2021). Sandbar willow (*Salix exigua*) plants decreased concentrations of single-ring and triple-ring diamondoid NAs in a hydroponic system within 6 h by 95% and 84%, respectively; for the single-ring NAs, the concentration decreased by < 20% within the first 30 min (Alberts et al., 2021). Various studies focused on the bioremediation of NAs in OSPW with CWTS using *C. aquatilis*, a locally common species to the oil sandss region, as it is a highly competitive species capable of tolerating tailings pond water, becoming the dominant species in these systems (Caners & Lieffers, 2014; Crowe et al., 2002). *C. aquatilis* has outperformed other tested species in reducing the toxicity of NAFCs in OSPW (Cancelli & Gobas, 2022; Crowe et al., 2002; Simair et al., 2021).

While field- and pilot-scale CWTS projects have shown they are a viable option for NAFC remediation (Ajaero et al., 2018; Simair et al., 2021), it is essential to gain a better understanding of how these passive, low-energy systems degrade OSPW (Cancelli & Gobas, 2020). Field studies are influenced by a large variety of uncontrollable environmental variables (e.g., rainfall events, seasonality, mechanical failure, and leakages) which lead to reduced experimental replicability, thus making conclusive observations difficult (Cappello & Yakimov, 2010; McQueen et al., 2017a, b; Simair et al., 2021). Mesocosm systems are built-in environments where factors that would be integral components in a CWTS can be controlled and evaluated (Cappello & Yakimov, 2010). Mesocosm systems create a representative exposure for test organisms while maintaining enough control to examine the effects of individual experimental parameters (e.g., OSPW typology, substrate, plant species, temperature), track biotic changes, and examine chemical dissipation pathways (Boyle & Fairchild, 1997; Schindler, 1998).

This study aims to evaluate the impacts of *C. aquatilis* on the attenuation of NAFCs in OSPW. Our study is conducted in a continuous surface flow CWTS constructed using sand and peat. The parameters examined within this mesocosm study will help to understand the different attenuation processes occurring in a CWTS.

## Materials and methods

### Experimental design

The experiment was set up in the Northern Forestry Centre’s greenhouse in Edmonton, AB. It consisted of three treatments with 4 replicates each: unplanted mesocosms with lab process water (LPW) not containing NAFCs, unplanted mesocosms with OSPW, and established *C. aquatilis* in mesocosms with OSPW. A total of 12 individual *C. aquatilis* plants was transplanted into each mesocosm prior to initiating the experiment. The mesocosms were randomly placed in the greenhouse bay.

The mesocosms were constructed out of polypropylene (50.8 cm height × 33.0 cm width × 129.5 cm length; 248.1 L) and were filled evenly with 10 cm (42.8 L) coarse sand tailings (CST) topped with 10 cm (42.8 L) peat–mineral mix (PMM). The CST and PMM represent locally abundant CWTS substrates and were collected from a mine located in the Alberta oil sandss. Either LPW or OSPW from the same mine was used to fill each mesocosm to 25 cm (139 L) above the substrate surface. The LPW was created to represent a similar chemical composition to OSPW excluding NAs to isolate the potential leaching of NAFCs from the substrate. The LPW was created using reverse osmosis water (RO) water containing 100 mg/L of calcium carbonate, 350 mg/L of sodium sulfate, and 100 mg/L of sodium chloride. The mesocosms were operated as surface flow closed loop systems, where the OSPW or LPW was pumped from a 159 L reservoir tank into the mesocosm. The pumps were set to cycle 28.8 L/day by pumping intermittently every 10 min, with a flow rate of approximately 20 mL/min. This flow rate sets each cycle to be 4 days (i.e., the amount of time required to exchange the entire volume of free water in the mesocosm with the reservoir) or a hydraulic retention time of 4.8 days; this experiment ran for 84 days or 21 cycles.

The *C. aquatilis* used in this study were grown from seeds collected from natural stands in seed zone DM1.3 (Dry Mixedwood). *C. aquatilis* seedlings were grown in a peat plug for 3 months in standard styroblock containers (size 512A). Seedlings were fertilized three times a week with Miracle Grow All Purpose water-soluble plant food (N-P-K ratio of 24–8–16). At the time of planting in the mesocosms, *C. aquatilis* plants were between 63 to 110 cm tall (mean = 83 cm). To acclimate *C. aquatilis* in the mesocosms prior to adding OSPW, RO water was used during the acclimation period (32 days) where the water level was slowly raised to achieve the final depth of 25 cm. The mesocosms were then drained and filled with 50% OSPW or LPW and 50% RO water for 7 days to acclimate the plants to OSPW, after which the mesocosms were drained and 100% OSPW or LPW was added to the desired depth of 25 cm. Throughout the experiment, the greenhouse temperature was maintained at approximately 20ºC with a 16-h daily photoperiod supplemented using LED grow lights. To account for evapotranspiration from the *C. aquatilis*, RO water was added to the reservoir tanks to maintain a consistent volume of water throughout the experiment.

General chemistry parameters for the substrate, OSPW, and LPW were analyzed at the beginning and the conclusion of the experiment. Substrate and OSPW samples were sent to Element Materials Technology (Edmonton, AB, Canada). The initial LPW samples were sent to the analytical lab at the Northern Forestry Centre (Edmonton, AB, Canada). Analysis for the substrates included pH, electrical conductivity (EC), sodium adsorption ratio (SAR), major nutrients, total petroleum hydrocarbons, and metal concentrations. Characterization of pH, EC, SAR, and major nutrients was determined using the saturated paste method, and metal concentration was completed using an ICP method with boron measured using hot water extraction. The analysis of OSPW and LPW included pH, EC, hardness, total dissolved solids, metals, major anions, and total alkalinity.

Various water, substrate, and plant parameters were measured at the end of every 4-day cycle. A YSI^®^ Professional Plus Multiparameter instrument was used to measure pH, dissolved oxygen, salinity, oxidation reduction potential (ORP), and conductivity of the water. Vegetation assessments included assessments of plant survival, vegetation cover, and height.

To determine the major ion concentrations for plant tissue, above-ground biomass for each mesocosm was collected and sent to Element Materials Technology for analysis (Edmonton, AB, Canada).

### Sample preparation and high-resolution MS analysis of NAFCs

Water samples were extracted using an ENV + solid-phase extraction (SPE) method, as has been previously described by Headley et al. (2002). SPE cartridges were rinsed with 6 mL of Milli-Q water, 6 mL of LC–MS grade methanol (Fisher Scientific, Hampton NH, USA), and conditioned with a further 6 mL of Milli-Q water prior to sampling. Sample aliquots were measured to 100 mL and acidified to pH < 2 with formic acid. Acidified samples were drawn through prepared cartridges at 1–2 mL/min under vacuum conditions, rinsed with 6 mL of Milli-Q water to desalt, and then dried under gentle vacuum. Sample extracts were eluted with 6 mL of LC–MS grade methanol then evaporated at 40 °C under a gentle flow of 5.0-grade ultrahigh purity N_2_ (Linde Canada, Saskatoon, SK). Dried sample eluents were reconstituted into 1 mL of 50:50 ACN:H_2_O with 0.1% NH_4_OH, then transferred to clean and labeled 2.0 mL LC–MS vials. Sample extracts were analyzed via loop injection to an LTQ Orbitrap Velos Elite™ mass spectrometer (Thermo Fisher Scientific, Waltham, MA) operating at 240,000 resolution (measured at 400 m/z) in negative ion electrospray ionization mode, as was previously described in the literature by Headley et al. (2011a). Concentrations of NAFCs were determined using a 5-point external standard calibration of Athabasca oil sands OSPW-derived NAFCs at known concentrations as described elsewhere (Ahad et al., 2020).

### Carboxyl group targeted carbon isotope analysis

Non-bitumen-derived organic matter such as fatty acids and humic material extracted with NAFCs can confound interpretations of naphthenic acid distributions in environmental samples (Ahad et al., 2013; Ajaero et al., 2017). Carbon isotope ratios of the CO_2_ generated by the pyrolytic decarboxylation (δ^13^C_pyr_) of NAFCs (also referred to as acid-extractable organics) were thus carried out to quantify the proportion of OSPW contributing to NAFC concentrations at three sampling points over the course of the experiment in the planted and unplanted OSPW mesocosms. The δ^13^C_pyr_ values were determined by thermal conversion/elemental analysis–isotope ratio mass spectrometry (TC/EA-IRMS) at the Delta-Lab of the Geological Survey of Canada (Québec, QC, Canada) using a Delta Plus XL isotope ratio mass spectrometer (Thermo Fisher Scientific, Waltham, MA) following a protocol adapted from Ahad et al. (2012). Around 1 L of water was collected on days 28, 41, and 84, acidified to pH 4.5, and extracted using loose Strata-X-A solid-phase extraction (SPE) sorbent (Phenomenex, Torrance, CA). The sorbent was then filtered from the aqueous phase under vacuum and NAFCs eluted with methanol containing 10% formic acid and pure methanol. The extracts were evaporated to dryness under ultrahigh purity N_2_ and re-dissolved in methanol. Small aliquots were transferred by syringe into a 40-μL rigid silver capsule, dried, and sealed with pliers prior to isotopic analysis. The δ^13^C_pyr_ values were determined using isotopically calibrated CO_2_ gas purchased from Oztech Trading Corp. (Safford, AZ, USA). Based on replicate standard and sample analyses, the uncertainty for δ^13^C_pyr_ values was ± 0.5‰.

### Data analysis and visualization

High-resolution mass spectrometry data were processed and background-subtracted in XCalibur software (version 2.2) (Thermofisher, 2011) and then imported into Composer64 software (version 1.5.6) (Paulssen & Gieg, 2019) to assign formulae from exact mass using a 3-ppm mass tolerance restricted to those containing H, C, N, O, and S. This non-targeted analysis workflow generates data with a level 4 identification confidence level (Schymanski et al., 2014). All formula data were imported into R Software (version 4.1.0) (Team, 2021) for wrangling and visualization. Plots were generated using ggplot2 (Wickham, 2016) or Microsoft Excel 2016 if the desired plot could not be achieved in R (Microsoft Corporation, 2018).

Prior to principal component analysis (PCA), base–peak normalized spectral abundance data were arranged, centered and Pareto-scaled (Ivosev et al., 2008; van den Berg et al., 2006), and then analyzed using the built-in R function prcomp() (Team, 2021). Where possible, figures were color-coded using colorblind-friendly viridis color palettes (Garnier, 2022).

To complete the analysis on the total NAFC concentration data, the *glmmTMB* package and the *tidyverse* packages were installed (Brooks et al., 2017; Wickham et al., 2019) and generalized linear mixed-effects models used to model the effect of treatments over time (intervals at Day 1, Day 40, and Day 83) on total NAFC concentrations (Bolker et al., 2009; Zuur et al., 2010). A generalized linear model was used to model the effect of the various soil and water parameters. Initial LPW parameters were not included in analysis computed in R since the parameters were collected from a different lab. The random effect that was used in the model was “mesocosm” to account for non-independent measures within each mesocosm. The *DHARMa* package was installed and used to complete model diagnostics (Harting, 2021), and the model with the best fit was used for visualization. A Gaussian identity link function was used for each of the following: total NAFC concentrations, CST (pH, arsenic, boron, chloride cobalt, lead, potassium, and nickel), and PMM (cadmium, chromium, cobalt, lead, nickel, vanadium, and zinc). Gamma log link function was used for each of the following: on all water parameters, CST (SAR, EC, calcium, sulfur, sodium, magnesium, vanadium, and zinc), and PMM (EC, pH, arsenic, barium, beryllium, boron, calcium, chloride, copper, potassium, magnesium, sodium, and sulfur). To test the fixed effects in models for their significance via the *Anova()* function of the *car* package (Fox & Weisberg, 2018), a Wald chi-square test was conducted on each model. Based on the fitted model, the estimated marginal means were calculated using the *emmeans* package (Lenth, 2018). The* P*-values for multiple-mean comparisons were adjusted using the Tukey method. Plant growth and plant health over time are presented descriptively to provide a general idea but were not analyzed statistically due to the low number of replicated samples.

## Results and discussion

### Substrate quality and characteristics

CST is a unique substrate that varies between operators due to the variability in ore type (Sarkar & Sadrekarimi, 2022). The CST used in this study was dominated by coarse sand (98% sand ± 0 (2 mm to 50 µm), 1.5% silts ± 0.6 (2–50 µm), 0.5% clay ± 0.6 (< 2 µm)). Trace metals, petroleum hydrocarbons (C10–C50), EC, and SAR in CST did not exceed any of the provincial soil guidelines (Table 1) (Government of Alberta, 2022).

**Table 1:** Coarse sand tailings physiochemical, inorganic, trace metal, and organic properties pre-experiment (initial) and post-experiment (final) per treatment; mean ± standard deviation. *P*-values and significant letters are based on generalized linear model

**Properties**	**Parameter**	***Initial***	***Final***			
		**CST**	**OSPW–unplanted**	**OSPW–*C. aquatilis***	**LPW–unplanted**	***P*-value**
		**(*n* = 4)**	**(*n* = 4)**	**(*n* = 4)**	**(*n* = 4)**	
Physiochemical	pH	7.2 ± 0.3^a^	7.7 ± 0.3^a^	7.8 ± 0.1^a^	7.6 ± 0.6^a^	0.110
	EC (dS/m)	0.8 ± 0.2^a^	0.7 ± 0.2^a^	0.9 ± 0.1^a^	0.9 ± 0.8^a^	0.743
	SAR	1.2 ± 0.1^a^	1.6 ± 0.3^a^	2.1 ± 1.1^a^	1.3 ± 0.5^a^	0.107
Inorganics (mg/kg)	Exchangeable Ca^2+^	30.9 ± 8.2^a^	29.5 ± 16.4^a^	34.6 ± 7.4^a^	55.8 ± 65.0^a^	0.479
	Exchangeable Na^+^	17.0 ± 2.8^a^	23.5 ± 2.1^ab^	31.3 ± 13.3^b^	19.8 ± 1.7^ab^	0.002
	Exchangeable K^+^	3.5 ± 0.6^a^	2.8 ± 0.5^a^	2.5 ± 2.4^a^	2.3 ± 0.6^a^	0.740
	Exchangeable Mg^2+^	8.2 ± 2.1^a^	8.2 ± 3.8^a^	10.4 ± 1.9^a^	13.5 ± 14.4^a^	0.596
	Exchangeable Cl^–^	3.0 ± 1.0^a^	11.0 ± 3.0^ab^	15.0 ± 8.0^b^	16.0 ± 4.0^b^	<0.001
	Extractable S	45.0 ± 12.0^a^	30.0 ± 21.0^a^	38.0 ± 10.0^a^	50.0 ± 79.0^a^	0.862
Trace metals (mg/kg)	Arsenic	0.6 ± 0.2^a^	0.7 ± 0.0^a^	0.7 ± 0.0^a^	0.7 ± 0.0^a^	0.633
	Boron	0.5 ± 0.1^a^	0.6 ± 0.2^a^	0.4 ± 0.1^a^	0.4 ± 0.3^a^	0.468
	Cobalt	0.9 ± 0.1^a^	1.0 ± 0.3^a^	0.8 ± 0.2^a^	0.8 ± 0.2^a^	0.388
	Lead	0.4 ± 0.1^a^	1.0 ± 0.3^b^	0.9 ± 0.3^b^	0.9 ± 0.2^ab^	0.001
	Nickel	1.2 ± 0.2^a^	2.4 ± 0.9^a^	1.6 ± 0.6^a^	1.6 ± 0.6^a^	0.050
	Vanadium	0.9 ± 0.2^a^	1.8 ± 0.9^a^	1.3 ± 0.5^a^	1.4 ± 0.5^a^	0.094
	Zinc	2.0 ± 0.0^a^	3.3 ± 1.3^a^	2.5 ± 0.6^a^	2.5 ± 0.6^a^	0.059
Petroleum Hydrocarbons	F2c C10–C16	82 ± 12	–	–	–	–
	F3c C16–C34	657 ± 82	–	–	–	–
	F4C34–C50 +	625 ± 91	–	–	–	–

The PMM used in this study had a lower SAR, EC, and cation exchange capacity than the general range of PMM used for reclamation in this region (MacKenzie & Quideau, 2012; Pinno et al., 2012; Schott et al., 2016), and the pH and trace metal concentrations were within the general acceptable range, meeting local guidelines (Table 2) (Government of Alberta, 2022; MacKenzie & Quideau, 2012; Pinno et al., 2012)*.* However, B increased substantially from the initial baseline concentration to the final concentration in the OSPW treatments, and the final concentration of B in PMM exceeded the provincial limit (Table 2) (Government of Alberta, 2022). It is hypothesized that OSPW is the likely source of boron in this experiment (Table 3). Boron adsorption in organic humic substrates is four times higher than clays or other substrates with little organic matter (Lehto, 1995; Parks & White, 1952). Therefore, it is reasonable to expect more B accumulated in the PMM compared to CST (Tables 1 and 2).

**Table 2:** PMM physiochemical, inorganic, trace metal, and organic properties pre-experiment and post-experiment with the various treatments; mean ± standard deviation. Bolded values exceed Alberta tier 1 guidelines (Government of Alberta, 2022). *P*-values and significant letters are based on generalized linear model

		***Initial***	***Final***			
**Properties**	**Parameter**	**PMM**	**OSPW–unplanted**	**OSPW–*C. aquatilis***	**LPW–unplanted**	***P*-value**
		**(*n* = 6)**	**(*n* = 4)**	**(*n* = 4)**	**(*n* = 4)**	
Physiochemical	pH	7.2 ± 0.1^a^	7.4 ± 0.2^b^	7.2 ± 0.1^ab^	7.2 ± 0.1^ab^	0.008
	EC (dS/m)	0.4 ± 0.2^a^	0.8 ± 0.2^b^	1.0 ± 0.5^b^	0.8 ± 0.1^b^	<0.001
	C:N ratio	29.5 ± 1.2	–	–	–	
	Total organic carbon (%)	5.5 ± 1.4	–	–	–	
Inorganics (mg/kg)	Exchangeable Ca^2+^	41.9 ± 20.5^a^	63.7 ± 23.9^a^	121.7 ± 114.7^a^	117.3 ± 76.0^a^	0.024
	Exchangeable Na^+^	2.8 ± 2.0^a^	116.8 ± 49.0^b^	99.5 ± 20.7^b^	164.3 ± 89.5^b^	<0.001
	Exchangeable K^+^	2.3 ± 1.0^a^	7.0 ± 3.4^b^	4.3 ± 2.6^ab^	5.3 ± 2.6^b^	<0.001
	Exchangeable Mg^2+^	10.8 ± 45.0^a^	17.6 ± 9.0^a^	27.6 ± 24.5^a^	25.9 ± 15.8^a^	0.071
	Exchangeable Cl^–^	4.5 ± 1.4^a^	61.25 ± 27.2^bc^	38.0 ± 8.0^b^	136.3 ± 87.4^c^	<0.001
	Extractable S	18.2 ± 18.5^a^	81.7 ± 53.4^ab^	119.5 ± 140.9^b^	83.3 ± 21.4^ab^	0.009
Trace metals (mg/kg)	Arsenic	4.0 ± 1.3^a^	3.8 ± 1.1^a^	2.8 ± 0.5^a^	3.1 ± 0.5^a^	0.091
	**Boron**	1.0 ± 0.2^a^	**9.1 ± 5.4**^c^	**4.7 ± 2.3**^bc^	2.0 ± 0.9^ab^	<0.001
	Barium	61.0 ± 9.6^a^	71.0 ± 14.4^a^	56.0 ± 6.2^a^	81.5 ± 42.7^a^	0.219
	Beryllium	0.2 ± 0.1^a^	0.2 ± 0.1^a^	0.2 ± 0.1^a^	0.2 ± 0.1^a^	0.695
	Cadmium	0.1 ± 0.0^a^	0.1 ± 0.0^a^	0.1 ± 0.0^a^	0.1 ± 0.0^a^	0.244
	Chromium	8.1 ± 3.2^a^	6.3 ± 2.2^a^	7.6 ± 1.2^a^	4.5 ± 1.4^a^	0.087
	Cobalt	3.6 ± 0.5^a^	3.7 ± 0.5^a^	3.2 ± 0.5^a^	3.1 ± 0.6^a^	0.295
	Copper	3.2 0.6^a^	3.9 ± 1.4^a^	3.2 ± 0.5^a^	3.4 ± 0.5^a^	0.476
	Lead	3.3 ± 0.6^a^	2.8 ± 0.3^ab^	2.7 ± 0.5^ab^	2.2 ± 0.7^b^	0.024
	Nickel	8.15 ± 2.3^a^	7.0 ± 1.36^a^	7.4 ± 1.1^a^	5.5 ± 1.4^a^	0.115
	Vanadium	11.4 ± 2.4^a^	9.5 ± 1.1^ab^	9.1 ± 2.3^ab^	6.8 ± 2.8^b^	0.018
	Zinc	13.5 ± 2.9^a^	16.3 ± 5.4^a^	14.8 ± 1.5^a^	14.0 ± 2.9^a^	0.643

**Table 3:** Lab process water (LPW) and oil sands process–affected water (OSPW) physiochemical, inorganic, and trace metal initial and final with the various treatments; mean ± standard deviation. *P*-values and significant letters are based on generalized linear model; initial LWP was not included in the statistical analysis because a different method was used to test those values

**Parameter**	**Initial**		**Final**			
	**LPW**	**OSPW**	**OSPW–unplanted**	**OSPW–*C. aquatilis***	**LPW–unplanted**	***P*-value**
	***(n* = *4)***	***(n* = *4)***	***(n* = *4)***	***(n* = *4)***	***(n* = *4)***	
pH	9.5 ± 0.02	8.0 ± 0.0^a^	8.5 ± 0.1^c^	7.7 ± 0.0^b^	8.4 ± 0.0^c^	<0.001
EC (dS/cm)	1.36 ± 0.02	1.23 ± 9.6^a^	1.20 ± 25.2^b^	0.74 ± 48.5^c^	0.90 ± 18.3^d^	<0.001
TDS (mg/L)	–	225.0 ± 3.8^a^	775.5 ± 18.5^b^	456.8 ± 34.4^c^	545.0 ± 19.3^d^	<0.001
Total alkalinity (mg/L)	7.8 ± 1.3	<5^a^	387.8 ± 30.1^b^	241.0 ± 16.1^c^	309.0 ± 23.0^d^	<0.001
Soluble Ca^2+^ (mg/L)	7.3 ± 0.3	54.4 ± 1.1^a^	83.0 ± 10.9^b^	51.8 ± 2.8^c^	70.38 ± 4.1^d^	<0.001
Soluble Na^+^ (mg/L)	267.5 ± 3.3	24.8 ± 0.1^a^	152.75 ± 5.9^b^	91.95 ± 8.3^c^	113.5 ± 3.5^d^	<0.001
Soluble K^+^ (mg/L)	–	190.0 ± 1.2^a^	9.9 ± 0.2^b^	0.7 ± 0.3^c^	1.7 ± 0.2^d^	<0.001
Soluble Mg^2+^ (mg/L)	–	53.5 ± 0.2^a^	30.4 ± 1.8^b^	15.1 ± 1.3^c^	15.3 ± 1.2^c^	<0.001
Soluble Cl- (mg/L)	157.3 ± 1.0	0.04 ± 0.02^a^	23.1 ± 1.1^b^	11.8 ± 1.2^c^	49.4 ± 0.9^d^	<0.001
Iron (mg/L)	–	13.7 ± 0.1^a^	0.02 ± 0.0^bc^	0.05 ± 0.0^b^	0.1 ± 0.1^c^	<0.001
Aluminum (mg/L)	–	0.1 ± 0.0^a^	0.02 ± 0.0^b^	0.004 ± 0.0^c^	0.05 ± 0.0^ab^	<0.001
Barium (mg/L)	–	0.2 ± 0.0^a^	0.05 ± 0.0^b^	0.04 ± 0.0^c^	0.04 ± 0.0^c^	<0.001
Boron (mg/L)	–	1.2 ± 0.0^a^	0.5 ± 0.0^b^	0.4 ± 0.0^b^	0.03 ± 0.0^c^	<0.001
Manganese (mg/L)	–	0.04 ± 0.0^a^	0.02 ± 0.0^a^	0.01 ± 0.0^a^	0.06 ± 0.0^a^	0.100
Strontium (mg/L)	–	1.1 ± 0.0^a^	0.4 ± 0.0^b^	0.3 ± 0.0^c^	0.1 ± 0.0^d^	<0.001
Vanadium (mg/L)	–	0.008 ± 0.0^a^	0.0008 ± 0.0^b^	0.0002 ± 0.0^c^	0.0004 ± 0.0^c^	<0.001
Zinc (mg/L)	–	0.008 ± 0.0^a^	0.007 ± 0.0^a^	0.01 ± 0.0^a^	0.005 ± 0.0^a^	0.271

### Water quality and characteristics

Dissolved oxygen concentrations remained above 5 mg/L throughout the experiment (Table 3) and generally varied with water temperature. During the experiment, ORP were measured between 10–20 cm depths, the values fluctuated between 60 to 200 mV across all treatments and remained positive during the entire study period (Table 3). The pH in the OSPW–*C. aquatilis* and LPW–unplanted treatments decreased over time compared to OSPW–unplanted treatment (Table 3), where it increased slightly. The LPW, which started with an alkaline pH due to the presence of calcium carbonate, reached equilibrium with atmospheric CO_2_ over time, leading to the stabilization of pH to around 7.2 (Table 3). The EC decreased across all treatment types (Table 3). The final general characteristics of the OSPW are similar to the findings of other studies (Cancelli & Gobas, 2020; McQueen et al., 2017a, b; Simair et al., 2021).

The presence of boron in the untreated OSPW (1.2 mg/L; Table 3), though only marginally below the provincial limit (1.5 mg/L)(Government of Alberta, 2018), showed a decrease after 84 days to 0.5 and 0.4 mg/L in the unplanted and the planted treatments, respectively.

### Naphthenic acid fraction compound dynamics

**Fig. 1:**
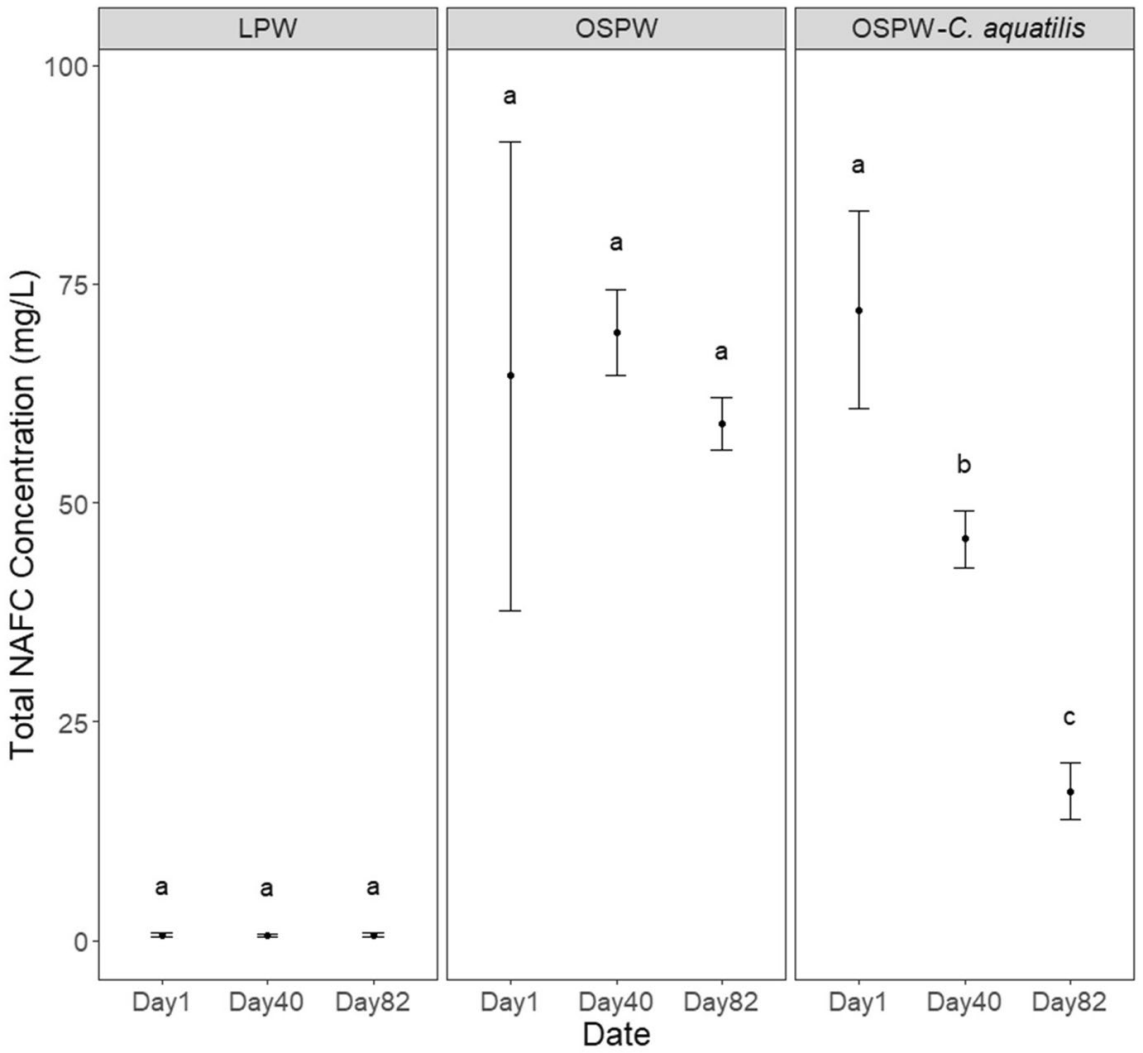
Mean total NAFC concentration (mg/L) in initial (day 1), middle (day 40), and final (day 82) per treatment. Error bars represent standard error for the means. Differing letters between means indicate a significant difference (*P* < 0.05)

Both quantitative and qualitative data describing NAFCs in the aqueous phase were collected throughout the experiment. Concentrations of NAFCs were dynamic, especially in OSPW-containing mesocosms. This is evident in the mesocosms with OSPW gradually reaching a maximum concentration after approximately 12 days, with gradually decreasing NAFC concentrations thereafter (Fig. 1). The concentrations of NAFCs were likely impacted by diffusion and adsorption during the first 12 days, dissipating afterwards in the remaining 72 days. The LPW–unplanted treatment had relatively little to no NAFCs with no significant change over time (*P* > 0.857) (0.6 mg/L initial to 0.6 mg/L final), suggesting minimal NAFC desorption from CST or PMM. After 84 days, concentrations of NAFCs in the planted mesocosms significantly decreased (*P* < 0.015) over time (72.1 mg/L initial to 17.1 mg/L final), whereas the unplanted treatments saw no significant decrease (*P* > 0.851) in total NAFCs over time (64.5 mg/L initial to 59.0 mg/L final) (Fig. 1). Between the OSPW–unplanted and OSPW–*C. aquatilis* treatment, there are large variations from the initial 4 mesocosm measurements. The initial (day 1) variation is likely because the OSPW has not had time to equilibrate within each mesocosm. There was a significant difference between treatments (*P* < 0.001) and a significant interaction effect from treatment and time (*P* < 0.001); however, timing of sample collection was not significant (*P* = 0.828) (Fig. 1). Further research into the influence of plants in conjunction with microbial community in the mesocosm will help identify the mechanism for the decrease of NAFCs.

The δ^13^C_pyr_ values in the unplanted mesocosms ranged from − 22.5 to − 19.5‰ and from − 21.8 to − 20.9‰ in the planted mesocosms (Table 4). Previous work has demonstrated a significantly more positive δ^13^C_pyr_ value for OSPW (− 20.7 ± 0.6‰) compared to non-bitumen, plant-derived NAFCs (− 27.9‰) (Ahad et al., 2013, 2020). The relatively narrow range of values calculated in both the unplanted and planted mesocosms thus indicates a predominantly OSPW-derived origin for NAFCs, with little contribution from non-bitumen-derived organics. Using a two end-member isotopic mass balance incorporating δ^13^C_pyr_ values for OSPW and non-bitumen contaminated background sources, the calculated proportions of OSPW in NAFCs were between 74–100% and 85–97% in the unplanted and planted mesocosms, respectively (Table 4). The results from δ^13^C_pyr_ measurements thus verify that the trends in NAFC concentrations shown in Fig. 1 and in the Orbitrap MS data, presented in subsequent figures, can be almost exclusively attributed to changes in OSPW-derived rather than plant-derived NAFCs, particularly during the latter stages of the experiment (i.e., days 41 and 84).

**Table 4:** The δ^13^C_pyr_ values and fractions of NAFCs derived from OSPW and non-bitumen sources calculated using an isotopic mass balance. The δ^13^C_pyr_ values for OSPW (− 20.7‰) and non-bitumen-derived NAFCs (− 27.9‰) used in the mass balance were taken from the literature (Ahad et al., 2020)

**Day**	**Mesocosm**	**δ**^**13**^**C (‰)**	**1σ (‰)**^**a**^	**f OSPW**	**f non-bitumen**
28	OSPW–unplanted	−22.5		0.74	0.26
41	OSPW–unplanted	−19.3		1.00	0.00
84	OSPW–unplanted	−20.7	0.47	1.00	0.00
28	OSPW–*C. aquatilis*	−20.9	1.00	0.97	0.03
41	OSPW–*C. aquatilis*	−21.8	1.00	0.85	0.15
84	OSPW–*C. aquatilis*	−21.1	1.42	0.94	0.06

^a^refers to the 1σ standard deviation between duplicate mesocosms. The error on δ^13^C_pyr_ measurements was 0.5‰

**Fig. 2:**
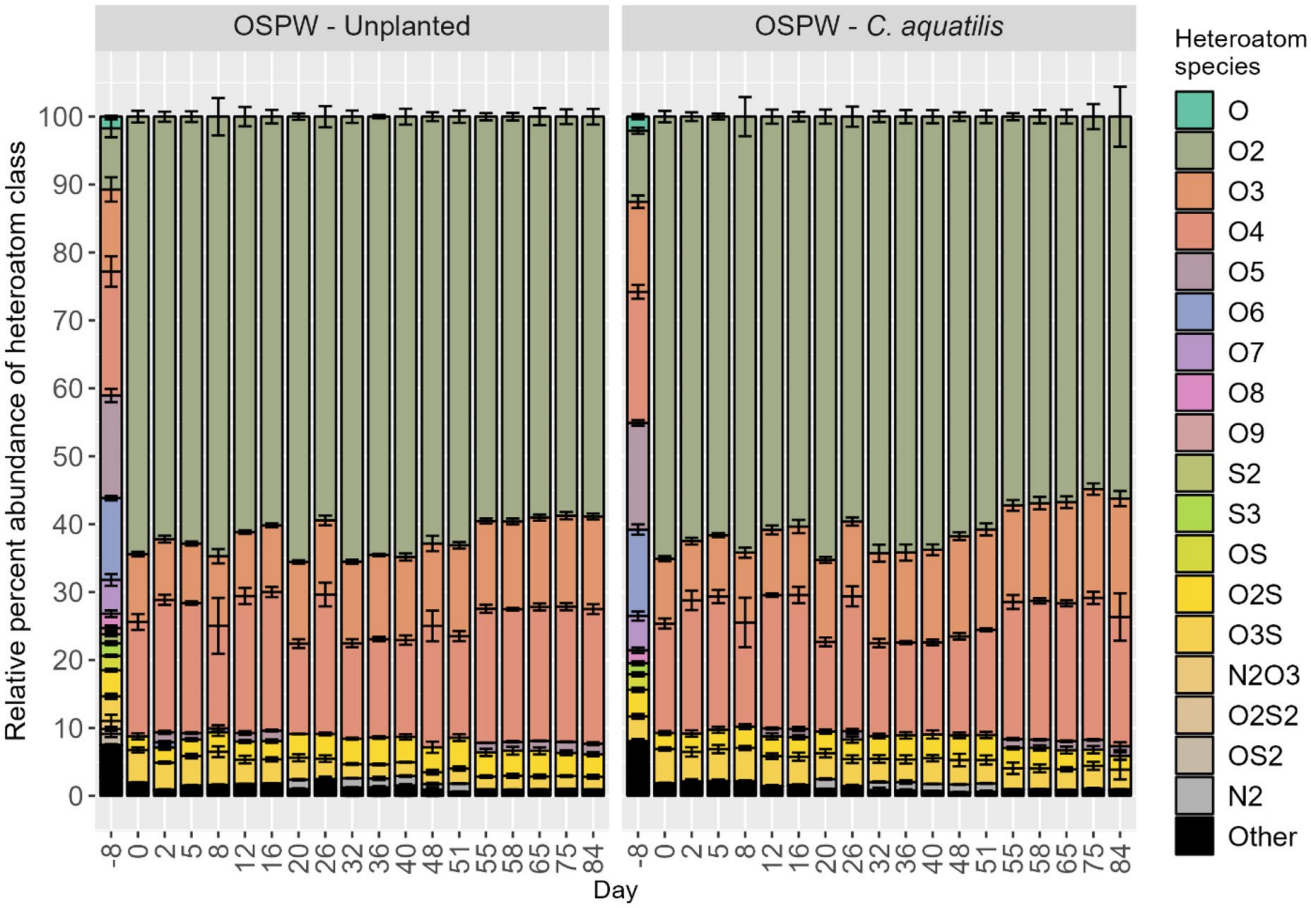
Percent-normalized heteroatom abundance of various formula classes, based on unique heteroatom inclusions (i.e., anything other than hydrogen or carbon), reported with standard deviation (*n* = 4)

To examine the changes in molecular formulae of NAFCs over the course of the study, data were organized according to unique heteroatoms (i.e., anything other than carbon or hydrogen) and plotted according to percent spectral abundance (Fig. 2). Sample data from the mesocosms with LPW were excluded from Fig. 2 owing to trace concentrations of polar organic compounds.

As compared to initial background conditions (i.e., day -8), the spectral signature of OSPW (i.e., high relative percent abundance of O_2_–NAFCs) remained strong throughout the 84-day treatment period in treatments with OSPW. Nonetheless, in both treatments, formula characteristics gradually shifted, where O_2_–NAFCs gradually decreased in relative abundance while O_3_– and O_4_–NAFCs gradually increased in relative abundance. The rate of decrease of O_2_–NAFCs (i.e., classical NAs) is almost negligible, decreasing by ~ 10% spectral intensity over the span of 84 days (Fig. 2). In a comparable study, the half-lives of selected NAs were estimated at 12–23 days (Ajaero et al., 2018). However, there are important differences between these two studies; Ajaero et al. (2018) used wetland mesocosms with upward vertical flow of OSPW through the entire growth medium, whereas the mesocosms in the present study operated with horizontal surface flow. Observable changes in surface water may be obfuscated by gradual intermixing and/or equilibrium with the OSPW in the pore water and the OSPW saturated porous media. The study by Ajaero et al. (2018) also used inorganic growth media (e.g., sand or gravel), making OSPW-derived organics the only available source of carbon, whereas the substrate used here included substantial organic-rich PMM, which may provide a favorable source of carbon for microbes. Further, Ajaero et al. (2018) used OSPW supplied by a different operator; accordingly, the typology of OSPW from their study and ours is not chemically identical (Frank et al., 2016), so it should not be expected to biodegrade identically.

**Fig. 3:**
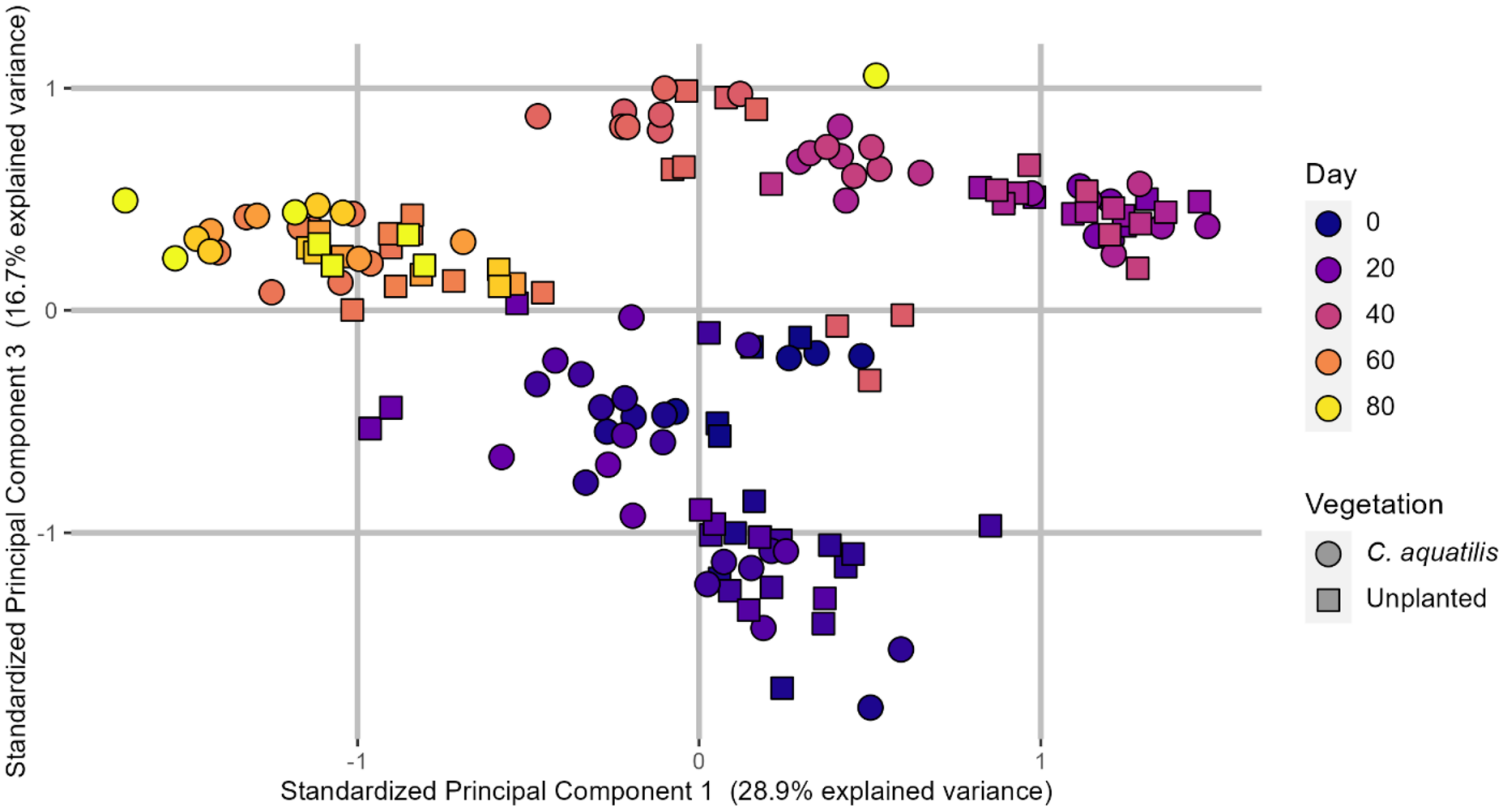
A principal component analysis (PCA) of all OSPW-containing samples, excluding lab process water

Although the bulk percent abundance of heteroatom classes remained relatively static, the spectra of certain mixture components changed over time. A gradual shift in mixture composition is supported by a principal component analysis (PCA) of all OSPW-containing mesocosm samples, shown in Fig. 3. Although sample data resolves incompletely during PCA, groupings of sample data from this experiment are best explained by sampling date. In contrast, there were no compelling differences between planted and unplanted systems when examined with PCA. The similarity between planted and unplanted systems likely could be th effect of adsorption to PMM. Further research into this hypothesis is currently underway.

**Fig. 4:**
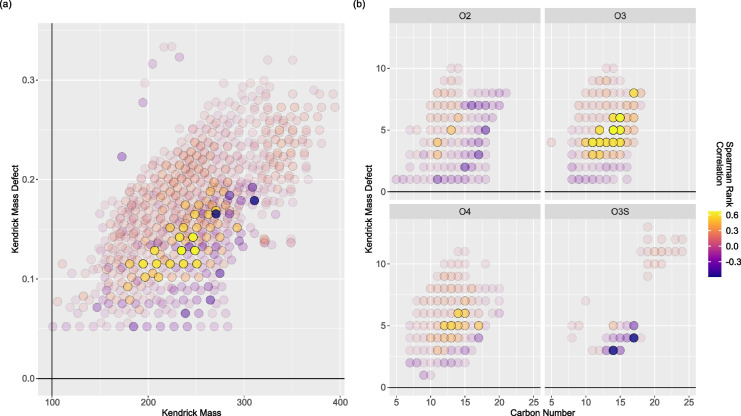
Plots summarizing spearman rank correlations of individual formula abundances using **a** Kendrick mass plot of all formulae detected across samples from OSPW-containing mesocosms, where this figure has been decomposed into major oxygen-containing formula heteroatom classes (**b**) that constitute approximately 95% of spectral intensity by carbon number and double bonding equivalents for clearer interpretation of trends. In both cases, scatterplot point transparency has been scaled by a factor of rho^2^ to highlight the strongest correlations

To examine the connection between sampling dates with the presence, emergence, or absence of particular formulae over time, the Spearman rank correlation between sampling dates and base–peak normalized relative formula abundances were calculated. The Spearman rank correlation was used to color-code a Kendrick mass plot and carbon number (#C) versus double bonding equivalents (DBE) plots in Fig. 4. High carbon number O_2_–NAFCs tend to have a low-to-negative correlation with sampling date, suggesting that many of these formulae, especially those with high molecular weight (i.e., > 225 m/z) and saturated formulae (i.e., KMD = 0.05–0.10), will diminish in relative spectral abundance over time. The two formulae with the most negative correlations from Fig. 4a are found in the O_3_S panel of Fig. 4b, corresponding to C_14_H_24_O_3_S and C_17_H_28_O_3_S. On the other hand, many O_3_–NAFCs have a considerable positive correlation with sampling date, suggesting that most of these formulae consistently increased in spectral relative abundance over time. The correlations of O_4_–NAFCs are moderately positive-to-neutral, suggesting that some of these formulae increased, while others persisted with unchanged abundance over the treatment period.

### Plant response

**Fig. 5:**
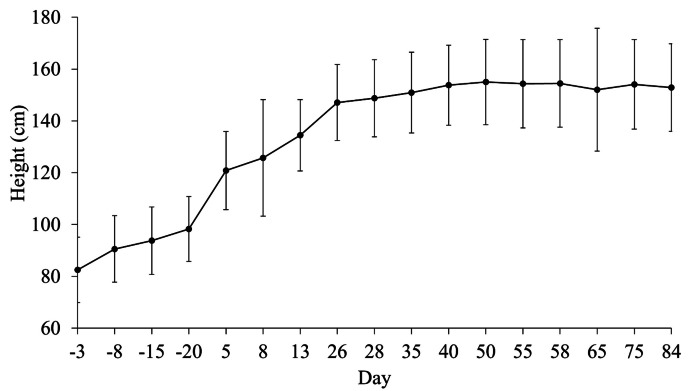
Mean height of *C. aquatilis* (*n* = 48) pre-experiment and during the experiment. Error bars represent the standard deviation

Throughout the experiment, OSPW–*C. aquatilis* had a 98% survival rate. Furthermore, all *C. aquatilis* individuals, apart from one, remained at 90–99% alive tissue through the experiment. However, all individuals in the OSPW–*C. aquatilis* presented some symptoms of chlorosis, necrosis, and/or mottling by the end of the experiment. Some individuals also presented signs of deformed and crinkled leaves. By day 40, *C. aquatilis* likely reached maturity with a growth threshold of around 150–154 cm in height (Fig. 5). Depending on the site conditions, the average height for *C. aquatilis* in Alberta is between 20 and 155 cm (Hauser, 2006; Johnson et al., 1995; Tannas, 2003; Vitt et al., 2020).

A majority of the ion concentrations were not notably elevated compared to other studies (Table 5). Although the mean manganese concentration in Table 5 is several orders of magnitude higher than that of other ions, the manganese concentrations in tissue generally vary between plant species (30–500 µg/g), making the results reasonable (Li et al., 2019; Millaleo et al., 2005). However, studies have shown toxicity effects in concentrations as low as 176 µg/g in *Juncus effusus L*. (soft rush) (Najeeb et al., 2009) and 150 µg/g dry weight in some crop species (Li et al., 2019; Millaleo et al., 2005). *C. aquatilis* in the mesocosm study started to show signs of toxicity expressed through chlorosis, necrosis, crinkling of the leaves, and mottling, which indicate reduced overall health over this 84-day study. It is unclear whether the decline in plant health was attributed to NAs in the OSPW or other ions in OSPW. Although commercial NAs are found to be more toxic than NAs derived from OSPW, Armstrong et al. (2009) found that systems with 30–60 mg/L of commercial NAs significantly reduced wetland plant growth. In the present study, the OSPW–*C. aquatilis* treatment had concentrations of 10.2–72 mg/L of NAFCs, of which 85–97% originated from OSPW. Further research needs to look at the concentration threshold for NAFCs in OSPW where toxicity effects are evident and the process of translocating NAFCS from the roots to the shoot tissues through the vascular system and how that relates to the concentration of NAFCs in plant tissues (young leaves, lateral shoots and roots, and root tips). Alberts et al. (2021) found that various NA groups are likely being partially bio-transformed when entering the central metabolism where the carbon from these compounds is assimilated into other macromolecules (e.g., lipids, starch, and cellulose). Although plant mortality and reduced growth in *C. aquatilis* were not observed here, further research is needed for improved analysis of NAs within plant tissue to more effectively evaluate potential toxicity effects from NAFCs (Alberts et al., 2021; Armstrong et al., 2009).

**Table 5:** The mean of above-ground tissue ion concentrations in *C. aquatilis* plant material (*n* = 4) with standard deviation

**Ions**	**Mean ± standard deviation**
Boron (µg/g)	22.4 ± 8.19
Calcium (%w/w)	0.7 ± 0.16
Copper (µg/g)	1.4 ± 0.30
Manganese (µg/g)	240.0 ± 80.71
Magnesium (%w/w)	0.3 ± 0.04
Molybdenum (µg/g)	2.9 ± 1.36
Nitrogen (%w/w)	1.0 ± 0.11
Phosphorous (%w/w)	0.2 ± 0.03
Potassium (%w/w)	1.2 ± 0.17
Sodium (%w/w)	0.2 ± 0.08
Sulfur (%w/w)	0.2 ± 0.04
Zinc (µg/g)	11.9 ± 1.99

## Conclusions

The present study reinforces the weight of evidence that CWTSs are a potential treatment method for OSPW as it is capable of attenuating NAFCs. Treatments with or without plants attenuated NAFCs in the water column according to similar distributions of molecular characteristics, though planted treatments seemed to outperform the unplanted treatments. Although results observed in this study showed NAFC attenuation in the water column to a lesser degree than previous work (Ajaero et al., 2018; Simair et al., 2021), important differences between the experiments likely contribute to the divergence in outcomes. First, previous studies used wetland mesocosms grown in mineral substrates (e.g., sand and/or gravel), with systems configured for vertical flow, and some systems included active aeration as well. These differences in study design likely impacted the distribution and/or degradation of NAFCs in the water column, namely, the adsorption and, by association, the temporary removal of NAFCs from the water phase to the organic substrate phase. This temporary removal conversely limited the availability of labile carbon in systems (e.g., system with no organic substrates) which could facilitate the degradation and assimilation of NAFCs for continued microbial growth. In addition, the vertical flow of water may enhance mixing across the water column, and increased aeration can maximize the potential for aerobic metabolism of NAFCs. In contrast, the mesocosm systems in the present study had surface flow and recirculation of OSPW over a porous mineral and organic medium, which in comparison could potentially adsorb considerable amounts of NAFCs temporarily. Slower diffusion and partitioning effects can obfuscate the rate at which transformation of NAFC species might occur, as noted in this study, where the period in which NAFCs changed the most rapidly occurred in the last 28 days of this study. This experiment also used OSPW from a single operator; it thus remains to be seen whether other OSPW types would undergo similar patterns of attenuation, further investigation into this is recommended. Past work has shown that gradual maturing and ecological succession in reclaimed wetlands can be associated with amelioration of toxicity therein (Armstrong, 2009, Cancelli & Gobas, 2020, Hendrikse et al., 2018; McQueen et al., 2017a, b. The effect of wetland age and related community maturity is therefore likely an important parameter that must be further evaluated to better understand relationships between ecosystem succession and weather of NAFCs.

## Data Availability

The datasets generated during and/or analyzed during the current study are available from the corresponding author on reasonable request.

## Abbreviations

OSPW: oil sandss process-affected water
LPW: Lab process water
CST: Coarse sand tailings
PMM: Peat mineral mix
NAs: Naphthenic acids
NAFCs: Naphthenic acid fractional compounds
CWTS: Constructed wetland treatment system
